# Identification of protein targets in red complex organisms binding with resveratrol

**DOI:** 10.6026/97320630016837

**Published:** 2020-11-30

**Authors:** Keshaav Krishnaa Pothapur, Veeraraghavan Vishnu Priya, Gayathri Rengasamy, Vijayashree Priyadharsini Jayaseelan

**Affiliations:** 1 Saveetha Dental College and Hospitals, Saveetha Institute of Medical and Technical Sciences, Saveetha University Chennai, Tamil Nadu, India; 2 Department of Biochemistry, Saveetha Dental College and Hospitals, Saveetha Institute of Medical and Technical Sciences, Saveetha University, Chennai, Tamil Nadu, India; 3 Department of Research, Saveetha Dental College and Hospitals, Saveetha Institute of Medical and Technical Sciences, Saveetha University, Chennai, Tamil Nadu, India

**Keywords:** Protein targets, red complex organisms, resveratrol

## Abstract

Periodontitis is attributed to the dental biofilm formation. Red complex organisms are a group of organisms linked with periodontal diseases. Therefore, it is of interest to identify potential targets from the red complex organisms to bind with the herbal compound resveratrol (E - 5 - (4 - hydroxy styryl) benzene 1,3 diol). We report a list of potential proteins having optimal drug like binding features with the herbal agent Resveratrol for further consideration. We used the STITCH v.5 pipeline VICMPred and VirulentPred tools to identify such targets as potential virulent factors in the red complex organisms. We considered the strains of Porphyromonas gingivalis ATCC 33277, Treponema denticola ATCC 35405 and Tannerella forsythia ATCC 43037 in the red complex pathogens for this analysis. Protein targets in the red complex organisms with optimal binding features with the herbal compound resveratrol were thus identified and reported for further consideration.

## Background

Dental biofilm or plaque can be expressed as the community of a range of microorganisms, which are found, on a tooth surface [1,[2]. The dental plaque is found to be one of the etiological factors for the development of gingival and periodontal diseases [3]. Periodontal diseases are polymicrobial in nature, which are an immune-inflammatory response to the presence of infectious diseases that can lead to the destruction of periodontal ligaments and adjacent supportive alveolar bone [4]. The subgingival plaque mircobiologically consists of over 700 bacterial species, and some of these microorganisms are to be held accountable for the initiation/progression of periodontal diseases [5,6]. The red complex pathogens include Porphyromonas gingivalis, Treponema denticola, and Tannerella forsythia (formerly Bacteroides forsythus), which are the most important pathogens involved in the development and progression of adult periodontal disease [7].

There are various treatment modalities that can be used for tackling gingival and periodontal diseases [8]. A few recent advancements include the use of local drug delivery systems and the use of ozone therapy [9, 10]. There has also been an evident rise in the practice of green medicine in periodontics through recent times [11]. Specifically, resveratrol is a well-known chemically and biologically active substance that is synthesised by plants when subjected to an insult such as infectious or ionising radiation and was pioneered by Renaud et al. [12]. As of today, there are about 92 new resveratrol compounds, which includes 39 dimers, 23 trimers, 13 tetramers, 6 monomers, 6 hexamers, 4 pentamers, and 1 octamer, all of these have been reported from the Dipterocarpaceae, Paeoniaceae, Vitaceae, Leguminosae, Gnetaceae, Cyperaceae, Polygonaceae, Gramineae, and Poaceae plant families [13]. Resveratrol is said to have very good antimicrobial and anti oxidative property which has been reported in existing literature [14,15]. Therefore it is of interest to identify potential targets from the red complex organisms to inhibit the herbal compound resveratrol.

## Materials and Methods:

### Study design:

**Figure 1:**
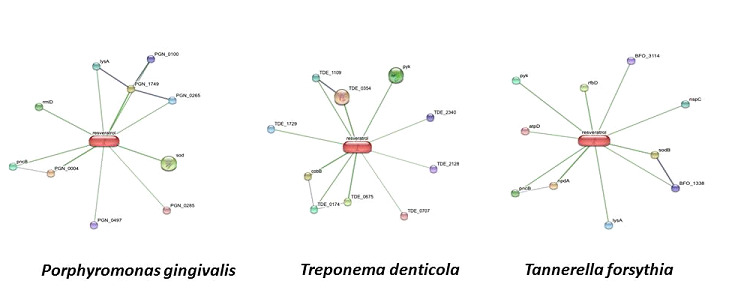
The STITCH v.5 pipeline analysis for the red complex organisms in target discovery

The present study follows the planning of an observational study, which primarily aims to screen for those proteins or virulence factors of red complex pathogens, which could possibly interact with resveratrol. The reaction as well as interaction of the compound with protein of bacteria was analyzed using STITCH v.5 pipeline [16] (Figure 1) and therefore the virulence properties of these interacting proteins were deduced and analysed by VICMPred [17] and VirulentPred softwares [18]. Porphyromonas gingivalis ATCC 33277, Treponema denticola ATCC 35405, Tannerella forsythia ATCC 43037 were the strains of red complex pathogens that were utilized in this study. These strains were included within the STITCH database, and therefore the query was user defined.

### Prediction of protein-drug interactions:

STITCH database (Version 5; 2016) is an extensive platform for various predicted or known interactions. It provides a comprehensive platform for known and predicted interactions between various compounds and proteins. The interactions between the compound and the organism could vary from direct or physical and indirect or functional associations, which primarily arise from computational prediction and from interactions aggregated from various other (primary) databases (Figure 1). The repertoire of proteins that interact with P. gingivalis, T. denticola and T. forsythia were further utilised for predicting virulence. [16]

### Virulence prediction:

VICMpred [17] and VirulentPred [18] pipelines were used for the identification of virulence factors targeted by Resveratrol among red complex pathogens.These tools employed support vector machine (SVM)-based five-fold cross-validation process to validate results. Virulence factors were screened on the idea of aminoalkanoic acid composition using VirulentPred tool, which classified them into two groups' namely virulent and avirulent factors. VICMpred categorises proteins into four major classes, such as, proteins involved in cellular process, metabolism, information storage, and virulence. The general potent accuracies of VICMpred and VirulentPred servers were 70.75% and 86%, respectively. The FASTA format of the actual proteins was retrieved from the NCBI database and was used as an input to run the algorithm [19].

### Prediction of subcellular localization of the virulent proteins:

The prediction of localisation of proteins at a sub cellular level helps in designing unique drug targets or for substantiating the role of an antimicrobial agent, which targets the virulent protein. Cell surface proteins are considered to be of great interest, as they will be used as vaccine targets. PSORTb V3.0 is an algorithm, which assigns a probable local site to a protein from an aminoalkanoic acid sequence that's provided [20].

## Results and Discussion:

The STITCH pipeline was used to identify the protein interaction between red complex bacteria and compound, resveratrol (Figure 1). Further each of the proteins interacting with the compound was assessed for their virulence property using VirulentPred andVICMpred. The scores produced by the algorithms confirmed the nature of the proteins and grouped them into two classes, virulent and avirulent. (Table 1 and Table 2 - see PDF). Proteins interacting with Resveratrol were primarily related to cellular processes, followed by metabolism and virulence factor. There were no proteins related to information storage that were identified. Interestingly, the scores from VirulentPred marked carboxynorspermidine decarboxylase and Superoxide dismutase Fe-Mn as virulent factors (Figure 1; Tables 1 and 2 - see PDF). STITCH prediction for resveratrol returned proteins mainly associated with metabolism and cellular processes. None for virulence factor and information storage were identified. Two compounds such as Pyridine nucleotide-disulphide oxidoreductase and hypothetical protein, associated with metabolism and cellular process respectively were found to be virulent based on the score obtained from VirulentPred (Figure 1; Tables 1 and 2 - see PDF). Out of proteins interacting with Resveratrol, majority belonged to Cellular Process, followed by metabolism and virulence factor. A protein, serpin associated with metabolism and a protein carboxynorspermidine decarboxylase were predicted to be associated with virulence. (Figure 1, Tables 1 and 2 - see PDF)

Evaluation of a particular compound is of utmost importance before the same has been tested for clinical practice. In helps us to acquire an accurate prediction of the results, which could be encountered while using the particular compound. It is particularly more cost effective when compared to in vitro evaluation. This method also provides more knowledge about the micro level activities such as pathways of actions and thus the compound can be better understood [21] There have been various herbal remedies [22] which have been developed in recent times to combat periodontitis, this is another attempt towards the same. There are various proteins, which have been found virulent in the case of Resveratrol. Resveratrol has been proven to have good Antimicrobial property and hence would be effective against these organisms [23]. There have been various invitro studies which have been conducted to prove the antibacterial property of Resveratrol and the lysis of bacteria occur either through Ring formation inhibition or gene expression [24] or through membrane alteration [25] The lysis of these bacteria could be through free radical or using anti oxidant property of resveratrol [26].

P. gingivalis is one of the most common bacteria associated with periodontitis [27] and the absolute elimination of the same as well as other red complex pathogens is rather difficult. The proteins from P. gingivalis that react with reseveratrol include, carboxynorspermidine decarboxylase and Superoxide dismutase. Carboxynorspermidine decarboxylase is found virulent in both P. gingivalis and T. forsythia hence targeted therapy will help to eliminate both the organisms. The present study is a one of its kind, which reveals several proteins, which reacts with resveratrol. Similar studies have also reported the effectiveness of phytocompounds against red complex pathogens [28]. More number of proteins of red complex pathogens reacts with resveratrol than with commonly used drugs such as acetaminophen and ibuprofen [29], which is, used more for symptomatic relief than therapeutic. However, the mechanisms, which lead to the lysis of these organisms, are to be confirmed with further in vitro investigations. There are a few limitations, which exist such as that the interaction could not have any functional significance and sometimes would not be able to reproduce the same in vivo.

## Conclusion

We report a list of potential proteins from the red complex organisms having optimal drug like binding features with the herbal agent resveratrol for further consideration.
